# The Impact of Gender, Nativeness, and Subject Matter on the English as a Second Language University Students’ Perception of Instructor Credibility and Engagement: A Qualitative Study

**DOI:** 10.3389/fpsyg.2021.702250

**Published:** 2021-06-22

**Authors:** Reza Rezvani, Parisa Miri

**Affiliations:** Department of English Language, Yasouj University, Yasouj, Iran

**Keywords:** instructor credibility, second-language instructor, engagement, nativeness, subject matter, gender

## Abstract

In instructional contexts, instructor credibility or ethos is deemed to play a paramount role in teacher–student interaction and relationships. Much effort has been devoted to instructor credibility conceptualization, measurement, and its association with other instructional variables of interest in dominantly quantitative inquiries. However, little research has been undertaken in second-language education in which communication is both a means and an end. This qualitative research set out to explore the perception of the students of instructor credibility in the context of higher English education and how gender, nativeness, and subject matter might impact their perceptions. It also aimed to study how instructor credibility could, in turn, influence the engagement and success of the students. Thirteen senior students of English as a foreign language from a university in Iran participated in this study. They were given a scenario about their prospective professors for two courses of “Research Methodology” and “Essay Writing.” The professors included four native English- and Persian-speaking male and female PhD holders. The participants were, then, interviewed about their perceptions of instructor credibility, their choices of instructors, and how they would affect their engagement. The data were recorded, transcribed, and recursively analyzed using an inductive thematic analysis. While instructor credibility is commonly characterized as a three-dimensional construct, involving competence, character, and caring, the data analysis generated a new component of performance concerned with the effectiveness of classroom knowledge presentation and activity organization. Caring also emerged as a constituent of a more inclusive component of rapport. Interestingly, albeit they viewed native English professors as generally more competent due to their nativeness, they perceived non-native professors as more credible for both courses, mainly because of their rapport building and familiarity with the needs and challenges of the students. Most of the participants also viewed male professors as more competent and communicative for both courses. The participants also tended to argue that perceived instructor credibility would encourage them to put in more effort in their academic undertakings and to engage in class activities. This would ultimately enhance their academic achievements and success. The paper discusses the findings and implications for second-language instructor credibility conceptualization and practice.

## Introduction

Persuasive communication and the impact of its source have been studied by rhetorical and communication scholars for several decades (Hovland et al., 1953; Andersen and Clevenger, 1963; Berlo et al., 1970; McCroskey and Young, 1981; McCroskey and Teven, 1999; Umeogu, 2012). Source credibility, or ethos in Aristotelian terms, emerged to study the attitudes toward the source of communication and persuasion, and how much credibility that source carries. Source credibility is, therefore, viewed as a significant facet of the communication process affecting the believability of the message sent by its source. Message persuasiveness and source credibility were conceptualized by Aristotle and Hovland et al. (1953), respectively, who contended that the credibility status of the source and the message efficacy and internalization are affected by the receivers’ perceptions of the source.

In the mid-1970s, the source credibility or ethos of instructors attracted the attention of the researchers in instructional contexts (Finn et al., 2009). It was deemed to play a paramount role in instructor–student interaction and learning outcomes. Credible instructors, as sources of change and influence in classrooms, arguably play a key role in developing and maintaining interest and engagement in teacher instruction and course work; uphold devotion to the principles of professional integrity and expertise; and display exemplary behaviors to students. An effective instructor produces significant social and instructional changes in the classroom and, in doing so, enhances the evaluation, interaction, and learning of the students (Fisher and Frey, 2019).

Instructor credibility generates from competent, caring, and reliable messages capable of impacting the perceptions, learning, and behavior of the students within educational contexts, and is associated with instructional communication behaviors such as affinity seeking (Frymier and Thompson, 1992), ego support (Frymier and Houser, 2000), assertiveness and responsiveness (Martin et al., 1997; Teven, 2001; McCroskey et al., 2004), expertness and verbal fluency (Myers and Bryant, 2004), argumentativeness (Schrodt, 2003), confirmation and clarity (Schrodt et al., 2006, 2009), and self-disclosure (Sorensen, 1989; McBride and Wahl, 2005).

According to Thweatt and McCroskey (1998), the fundamental aim of classroom communication is to generate understanding by engaging both the affect and cognition of the students. Instructional communication is then investigated in terms of relational and rhetorical theories (Mottet and Beebe, 2006). The relational approach to communication is grounded in the reciprocal, cooperative nature of communication or interaction between students and instructors, who make an equal contribution to the classroom instruction and enhance shared knowledge and cocurricular opportunities. In contrast, in rhetorical communication, instructors are the primary senders of classroom messages as well as the sole creators of the meaning and experience, informing instructional practices and learning direction. This model accounts for a rather linear process and passive learning in which learners are the receivers of the messages and instructions (2006).

Instructor credibility theoretically bears affinity with the rhetorical approach to communication since instructors are the social persuaders and communicators of knowledge and meaning. Increased perceived instructor credibility is then dependent on the transfer of information (McCroskey et al., 2004). However, more recently, examining perceived instructor behavior and effectiveness, Myers et al. (2017) suggested that instructor credibility is both rhetorical and interpersonally driven, that is, the content delivery and task management of the instructors are viewed as rhetorical by students, whereas his/her character and personality traits, such as empathy and sociability, are assumed to be part of relational behaviors that affect interpersonal communication.

Despite having revised the source dimensions or conceptualizations by adding a new component or rewording the existing ones, researchers have generally agreed that the two dimensions of competence and trustworthiness are pivotal to perceptions or views of credibility (McCroskey and Teven, 1999). This is because the two constructs of trustworthiness and caring behaviors are deemed to represent the interpersonal communication and involvement of the instructors (Myers, 2004). Over the past decades, underscoring credibility in classroom communication and learning instructional scholars have also persistently attempted to dimensionalize credibility (Finn et al., 2009; Schrodt et al., 2009). The credibility components were thought to involve intelligence, character, and goodwill according to Aristotle, and expertness, trustworthiness, and intention toward the receiver as conceptualized by Hovland et al. (1953). In one of the earliest experiments, McCroskey et al. (1974) developed five dimensions of source credibility, including “composure,” “extroversion,” “competence,” “character,” and “sociability,” although acknowledged that the two latter credibility components are likely to be conjoined.

Later in 1981, McCroskey and Young (1981) disputed the multiplicity of the source dimensions and even contended that attempts at dimension development minimize the objectivity of the findings. They proposed that there are three underlying constructs of source credibility, however, when a factor analyzed, “goodwill” overlapped with the two dimensions of competence and trustworthiness, and ultimately collapsed into one of these two stand-alone constructs. This conceptualization was also later challenged, owing to the measuring instrument and methodology employed and revisited by McCroskey and her colleagues. They maintained that goodwill, which concerns the welfare, educational, and psychological needs of the students, merits isolation from the two constructs of competence and trustworthiness (Teven and McCroskey, 1997; McCroskey and Teven, 1999). They also emphasized the impact of goodwill in increasing communication skills, personal involvement, and the cognitive and affective learning of the students. This was also confirmed by Finn et al. (2009), contending that perceived caring importance and contribution dwarf those of other source credibility constructs. Caring instructors, according to McCroskey (1992), communicate openly with students, welcome their unsuccessful attempts and errors, assume the best in the students, and, overall, are understanding, empathic, and responsive. In other words, “students certainly are going to listen more attentively to a person who they believe cares about them and has their best interests at heart” (p. 110).

Thweatt and McCroskey (1998) reconceptualized the credibility model and added two new components of dynamism and immediacy in addition to trustworthiness and competence. They argued that dynamism signals instructor passion and enthusiasm for the subject matter, and immediacy signals the psychological and social closeness of the instructors. They also found that an instructor who is perceivably immediate, albeit less able, communicates more concern and caring to students than an accomplished instructor who is non-immediate and concluded that immediacy mediates the credibility perceptions of the students and produces desired learning outcomes.

McCroskey and Teven (1999) developed an 18-item semantic referential scale to measure the perceptions of the students of instructor credibility and demonstrated that competence, character, and caring construct instructor credibility. This classical study and three-partite conceptualization are often drawn upon and most cited in instructor credibility research (Finn et al., 2009). Drawing on this credibility characterization, Myers and Bryant (2004), for example, found the content expertise and verbal fluency of the instructors indicate the competence of the instructors; their integrity, immediacy, flexibility, and understanding indicate their character; and their responsiveness, accommodation, and accessibility convey their caring.

However, other studies tended to limit the construct of credibility to one or two components influencing instructor credibility status or image and views of the students. For example, a qualitative study by Hendrix (1997) indicated that the single component predicting and mediating instructor credibility in the eyes of her students was competence, comprising subject matter knowledge, work experience, good teaching techniques, and clear instructions. In her auto-ethnographic study of the perceptions of the students of foreign instructor credibility, effectiveness, and communication, Zhang (2014) also reported rapport and competence as dimensions leading to improved instructor evaluation and a positive classroom climate conducive to learning. She further highlighted that good rapport is associated with those interpersonally driven communication behaviors, including understanding, openness, role modeling, and appropriate use of space, whereas competence concerns content-based dimensions represented in subject expertise, presentation clarity, and relevant knowledge. Subscribing to two-dimensional instructor credibility, Chory (2007) maintains that responsiveness, accommodation, and accessibility characterize instructor caring, while affinity-seeking behaviors, flexibility, trustworthiness, and understanding define perceived instructor character.

Credibility research, including instructor credibility, has been much centered around attempts to decompose the construct and its components. More recently, scholars also have turned their attention to how the components are impacted or assisted by other instructional variables of interest. For instance, some scholars (e.g., Paige, 1990; McCroskey, 2002, 2003; Meyer and Mao, 2014; Punyanunt-Carter et al., 2014; Pishghadam et al., 2021) examined perceptions of credibility in terms of the nativeness and gender of the instructors, as the two variables of interest in this study. Interestingly, they suggested that domestic instructors received higher evaluations than foreign instructors. Cross-cultural studies of the credibility of native and non-native instructors (e.g., Rojas Gomez and Pearson, 1990; Neves and Sanyal, 1991) have also revealed that students rated domestic instructors more highly in terms of character and communication while perceiving intercultural instructors as more competent. Thus, students had a tendency not to sign up for classes conducted by international instructors (McCroskey, 2002). Furthermore, some researchers noted that the expertise and immediate behavior of the instructors are more likely to affect the judgments and learning of the students than variables such as their gender and nativeness (McCroskey, 2002, 2003; Glascock and Ruggiero, 2006). Therefore, if students’ learning and perceptions are lowered, it is presumably due to communication breakdown, resulting from language barriers and less speech clarity of the instructors (McCroskey, 2002, 2003).

In view of gender, students were found to perceive female instructors as reflecting more on students’ milieu and, therefore, higher in character and credibility than male instructors (Rojas Gomez and Pearson, 1990). Conversely, in the study of Hargett (1999), male instructors were rated more highly and thought of as more credible than female instructors. To confound the picture, in the study of Statham et al. (1991), female and male instructors were rated equally. Perhaps, the inconclusive findings are due to the role of a multitude of contextual factors at play in instructional context, resulting in different perceptions by the students.

Instructional studies on credibility have also had an intrinsic motive to see the influence of perceived instructor credibility on classroom learning and course outcomes of the students. Instructor credibility can improve students’ quality learning indicators and desired outcomes such as increased motivation to learn (Frymier and Thompson, 1992; Martin et al., 1997; Tibbles et al., 2008), higher affective and cognitive development (Teven and McCroskey, 1997; McCroskey et al., 2004; Tibbles et al., 2008; Finn et al., 2009), willingness to talk (Myers, 2004), and self-efficacy (Won et al., 2017).

McCroskey et al. (1974) examined the hypothesized association between instructor credibility and the ability to recall information as a learning outcome. Their experiment suggested that competent instructors could provoke and improve the attention, memory, and recall of the students. They, in addition, found that instructor temperament as an instructor communication behavior affects the credibility evaluations of the students and their academic achievement. Furthermore, credible pedagogues, who are immediate, homophilous, and passionate about the course content and instructions, play a considerable role in student persistence in and commitment to the academic studies and undertakings (Wheeless et al., 2011). The support that instructors provide also fosters learning and student–instructor communication (Schrodt et al., 2009). Students whose instructors are nonverbally immediate are unlikely to miss their classes (Rocca, 2004) and might even take additional courses (Witt and Wheeless, 2001). They also tend to participate and engage in classroom discussion and conversation, and solicit information when they perceive their instructors as attractive, respectable, and extroverted (Wheeless, 1975).

Although instructor credibility research and theory are rich in conceptualization and measurement and have seen much effort in exploring their association with other instructional variables of interest, the approaches taken have been dominantly quantitative (Hendrix, 1997; Finn et al., 2009). Few studies have also been undertaken in second-language education in which communication is essentially both a means and an end. This qualitative research set out to explore the perceptions of the university students of instructor credibility in the context of higher English education. More specifically, it is intended to study how the gender and nativeness of the professors, and subject matter might impact on English as the perceptions of the second-language (EFL) university students of the credibility of their instructors. It also aimed to study how instructor credibility could, in turn, influence the engagement and success of the students. The study addresses the following research questions:

RQ1: How is instructor credibility perceived in the context of university EFL courses?

RQ2: How is perceived instructor credibility in the context of university EFL courses affected by an instructor’s nativeness, gender, and subject matter?

RQ3: How does perceived instructor credibility affect university EFL students’ academic engagement and success?

## Materials and Methods

### Participants

From among 30 Bachelor of Arts (B.A.) EFL students, 13 students consented to participate in the study. They were all Persian-speaking senior students from a university in Iran in the first semester of the academic year 2020–2021. They were four males and nine females with an age range of 22–29. All the participants had taken courses in which they covered and discussed language teacher characteristics, including instructor credibility.

### Data Collection

This study reports the findings from a larger study on the credibility of the EFL instructors from the point of view of postgraduate and graduate EFL students. When designing the study and, particularly, the interview questions, we had two options, that is, using the same general questions for both groups or developing more specific and contextualized questions for the graduate students. Since we did not expect the graduate students to give us expert views and that we sought to elicit their contextualized perceptions of EFL instructors, we opted to design a scenario for the interview questions. More specifically, the interview questions were based on the scenario situating the research questions in an EFL university context (see Appendix A). This helped us to generate more context-specific themes. The scenario described their prospective professors for two two-credit courses of “Research Methodology” and “Essay Writing” from among required courses from the program. We deliberately selected these two courses, which are intended to develop a subject matter area and English proficiency, respectively. The professors also included two native English (male and female) and two Persian-speaking (male and female) PhD holders. We asked them to choose the instructors for these two courses and then tell us why they preferred them and how they perceived their credibility.

The interview questions were developed based on a review of instructor credibility literature and in line with the research questions. The scenario and interview questions were both piloted with two students from the same class. They were requested to respond to the questions and also comment on the clarity of both the scenario and the questions. This piloting led us to amend some wording for more clarity. Interviews were conducted individually over the telephone, taking no more than 25 min. Prior to the interviews, the students were given a copy of the scenario so that they could refer to the information in it during the interview.

### Data Analysis

The interviews were recorded, transcribed, and recursively analyzed using an inductive thematic analysis. The analytic process was followed in three basic steps of data familiarization, code generation, and theme extraction (Braun and Clarke, 2006). During the initial phase of the analysis, the interview data were transcribed verbatim and studied thoroughly for issues or views of interest. Following this, several tentative coding categories emerged, which were then constantly examined and revised when new codes or discrepant instances were encountered (Tesch, 1990; Glaser, 1992; Strauss and Corbin, 1998). Emergent codes were then juxtaposed and drawn together to generate underlying themes. The identified themes were organized and interpreted by cross-referencing among and across the transcripts. The themes which summarized the narratives of the participants, along with some exemplary perceptions, were incorporated into the final report.

In order to enhance the trustworthiness of the findings, several steps were taken. To ensure the accuracy of the coding and to reduce bias or uncertainty, field notes were also taken while interviewing the participants. In addition, memos were recorded and referred to for more precise data analysis (see Saldaña, 2011). In order to ensure data coding and analysis consistency, the interview data were first coded and analyzed by the second researcher, and a portion (20%) of it was reanalyzed by the first researcher. Inter-coding reliability was initially 85%. Areas of disagreement were discussed and resolved, followed by a reanalysis of a new portion (10%) by both researchers, which resulted in an agreement rate of 96%. The data were then analyzed again by the second researcher for any amendments. As recommended in qualitative research (for more details, see Lincoln and Guba, 1985; Creswell, 1998), the findings and the interpretations made were returned to a couple of the students for member checking (Harvey, 2015) to see whether they resonated with their perceptions.

### The Study Findings

In what follows the key findings, along with some illustrative comments and quotes, are presented and discussed in relation to the research questions.

RQ1: How is instructor credibility perceived in the context of university EFL courses?

**Figure 1:**
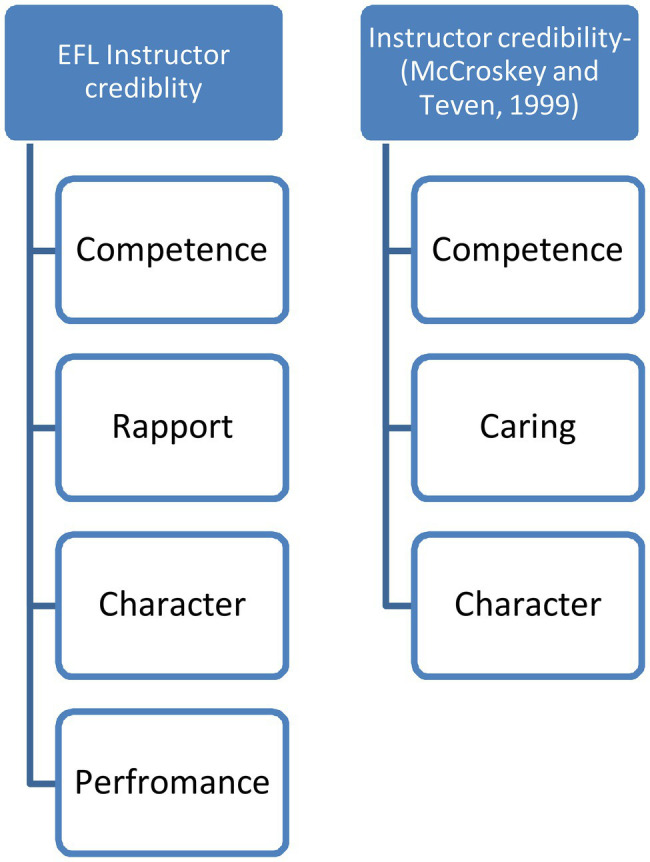
Instructor credibility conceptualizations.

The first research question concerned the perceptions of the students of the characteristics and skills of a credible instructor in the context of university EFL courses. Four key components or themes, along with several subcomponents of the credibility construct, emerged. The results revealed that a credible EFL instructor, in the eyes of the student participants, possesses both professional and linguistic knowledge, is able to establish rapport with students, has an appealing character, and can organize and present knowledge and skills well. Figure 1 depicts the instructor credibility framework developed in this study and the one advanced by McCroskey and Teven (1999).

Competence was perceived as the most significant indicator of the instructor’s credibility. The most prevalent theme that the students expressed in relation to the first research question comprised professional or linguistic knowledge. Describing a credible instructor, Zeynab, for example, stated that the greater the knowledge, the greater the trust. For some students (e.g., Arash, Zohre, and Fatemeh), only competent instructors have the knowledge and ideas to communicate with confidence. Competent instructors “know what they are doing,” Fatemeh argued. She also commented that credible instructors can always create and foster knowledge and communication in the classroom by stimulating the participation and contribution of the students. The students repeatedly pointed to a “broad knowledge base,” “vast competence and competency,” and “expertise” when describing credible EFL instructors. We asked the respondents to further characterize such general attributes of credible instructors.

What they additionally expressed included content knowledge, pedagogical knowledge, general knowledge, and knowledge of both languages (L1 and L2). For Arash and Zohre, an instructor “should have deep knowledge of the subject” in order to stimulate the positive perceptions of the students and earn their trusts; otherwise, as commented by Ali, “I do not ask my questions…I do not seek help when I have a problem in my course.” Zahra added that a credible instructor has both content literacy and general knowledge, which are required to integrate current content with prior knowledge within and across the subjects and to relate what they learn from the real-life experiences of the students. Another salient and prevailing comment of the EFL respondents was the competence and mastery of an instructor of L1 and L2. Zohre, for example, commented that an English instructor “needs to be fluent in both languages and be able to make comparisons between them.” She interestingly maintained that “Since we are always instructed by non-native instructors…they have got to know both the English and Persian literature, speak with fluency and accuracy to first convince us that they themselves know what they teach” “to be dependable.”

In addition to the types of knowledge, students also viewed the quality of knowledge communicated by the language instructor as significantly affecting the teaching quality and their perceptions and evaluation. The qualities encompassed clarity, adequacy, and currency. The subject matter or course content is, according to Arash and Ahmad, to be communicated clearly through the effective communication and proper teaching procedure of the instructors. The participants perceive instructors highly when they possess the knowledge and are able to communicate it clearly. This is particularly evident when they can provide satisfactorily clear and adequate answers to questions or solutions for problems. Interestingly, when they refer students to reliable sources when teaching or for further study, the students take it as evidence that their instructors are also reliable for they are current and aware of academically reliable resources, Zohre commented.

The second credibility component emerging from the data is rapport of instructors. This perceived instructor rapport, indicating mutual understanding and compromises between the instructor and the students, exemplifies communicative qualities such as caring, empathy, encouragement, and immediacy. In the eyes of the participants, interpersonal interaction and communication of instructors play a very important role in their credibility. One aspect of this social interaction involves caring. Credible instructors “care about students’ emotional well-being, intellectual needs and concerns” and “are willing to listen attentively to what we [they] have to say,” Shahla stated. For Zohre, instructors’ asking students about their problems, questions, and comments or suggestions is an indication of the instructors caring about them and their learning.

In addition, credible instructors are perceived as empathetic and understanding of struggles and progress of students, particularly when learning a foreign language. Emphasizing instructor rapport and understanding, Maryam recalled that some instructors might just evaluate assignments and class performance of their students to shape their communication with the students. In comparison, she reasoned credible instructors empathetically understand psychological or emotional state of their students and know that classes and learning, particularly in a new language, may involve tension and anxiety, which reduce when they are heard, negotiated, or dealt with properly. A repeated theme we noted from the comments, although we did not ask them about the consequences of lack of understanding of instructors, was that it may not only erode perceptions of students of rapport and credibility of the instructors but also affect their interest and motivation to continue learning. In words of Maryam, it might make an instructor “to fall from grace.” Finally, students also assessed encouraging and immediate behaviors of instructors and their association with credibility. In view of the participants, a highly immediate instructor “creates a feeling of comfort and intimacy in the classroom,” “feels more connected,” and “feels he/she is one of us” (Shahla and Ali).

Responses of students also generated a third credibility component of “character” concerned with personal characteristics and behavior patterns of instructors, affecting their credibility. A credible instructor is ethical, possesses a good character, and values students and does not humiliate them, argued by Maryam and Ahmad. Morality, thus, does have a significant impact on instructor credibility, “no matter what their academic ranks or professional statuses are,” Maryam commented. She also added that “I do not mind whether he’s well-educated or not, an assistant or associate professor…as long as he does not show a moral character, he is little credible.” This perceived character, in further analysis, comprises multiple qualities such as courtesy, realisticness, humor, and enthusiasm.

Perceived courtesy plays a significant role in inspiring interest and respect of students, developing an appreciation of the course content and the instructor, and promoting their motivation to learn and remain active throughout the course. This instructor courtesy or respect, in Zahra’s view, also fosters “a relationship of trust between instructors and students.” Perceived realisticness was also deemed as important in increasing perceptions of students of instructor credibility. Some students like Fatemeh complained about the unrealistic and heightened expectations demanding them “to bite off more than [what] we can chew,” which unfavorably affects their perceptions of the character of the instructor. Another important facet of credible character of instructors was their sense of humor. This, according to some of the students like Negin, makes learning fun, reduces fatigue and anxiety in language learning classes, to the extent that “it can compensate for inadequacies in knowledge and performance of an instructor.” A relevant attribute arising from the analysis was the passion or enthusiasm of instructors for teaching and for classes which attracts “positive attitudes of students toward the instructor and the course,” Fatemeh commented.

The last data-driven component is the classroom “performance” of the instructors or their ability to organize and present activities, personalize learning to meet varying needs of students and interact well with the students. A common theme was that those who hold a professorship at a university must have met the acquired competence and skills, and what makes a difference is their actual in-class performance. According to Mohamad, instructors need to be able to put to use what they learned in theory, or, in the words of Fatemeh, instructor credibility is not just a question of “what” but also of “how.” Students will be increasingly disappointed if their instructors fail to set up and organize various instructional activities, assignments, and discussions to convey knowledge and skills. In other words, the teaching of instructors will demonstrate and foster instructor credibility in the long run. Ali indicated that, since he is a student of language teaching, he usually waits to “see how the instructors teach through the term” and whether “they practice what they preach” to evaluate them. Initial perceptions of instructors, positive or negative, might change through actual classroom observation of what instructors “do, and not pretend,” Maryam stated. It is worth noting that there were variations in the perceptions of the respondents of other components of instructor credibility; however, when it came to this aspect, they rather unanimously pointed to or implied the importance of performance.

RQ2: How is perceived instructor credibility in the context of university EFL courses affected by an instructor’s nativeness, gender, and subject matter?

The second research question probed the preferences of the EFL students in relation to their perceptions of instructor credibility. Since the scenario leads them to think of the prospective instructors in terms of nativeness, gender, and subject matter, the emergent themes are presented in relation to these variables. When we were designing the scenario, we thought that most EFL students would probably select native speakers of English for “Essay Writing,” but we had no idea as to how many of them would opt for non-native (Persian-speaking) instructors for “Research Methodology” courses. However, only two students (Shahla and Zohre) preferred native instructors for both courses because of their competence while the majority (n = 11), although with the same view about the higher competence of the native instructors, opted to take both courses with non-native instructors for their rapport with the students and their understanding of the needs and challenges of the students.

As for writing in English, native instructors of English were generally deemed to have sophisticated and in-depth competence engendered by first-hand and long exposure to English as their mother tongue and longer experience in English writing. They have to write in English, “so practice made them perfect,” as commented by Ali. The vast knowledge of English enables native instructors to discuss and cover the subtleties of Essay Writing, which might be impossible for a non-native instructor to acquire and teach. This competence superiority, particularly in English proficiency, led only two students to prefer native instructors. Zohre and Shahla were also concerned about international examinations like IELTS and TOFEL. They heard that the writing section of such tests is demanding, and native instructors could be very helpful. For the same reason, they preferred native instructors. Also, assuming that such teachers are competent and expert in subject matters, they maintained that they could draw on up-to-date and effective teaching methods to enhance the knowledge and skills of the students. When justifying her preference, Shahla recalled having watched instructional YouTube videos, demonstrating native English instructors teaching international students by incorporating “real-life examples” and “hands-on experiences” that she valued much.

Regardless of the competence and language proficiency of the native instructors, the other students evaluated the credibility of the non-native instructors more highly for both courses because they were thought to be aware of and sensitive to the educational and psychological needs and problems of the EFl learners. Mohamad regarded non-native instructors as more caring and capable of relating to students well because “they have gone through the same things.” These instructors, according to the students like Nahid and Zahra, are more likely to demonstrate empathy with the needs and problems of the students because they live in the same community, and have experienced the same cultural and educational norms, expectations, and standards. In doing term research and class English essays, they were viewed to know and understand their limitations as students of a foreign language. Informed of the recurrent problems in the EFL context, they might not “expect too much and can help us,” Shima contended. Sometimes, “even if you do not say anything, non-native instructors can understand you and your mood from your eyes and face,” stated Shima.

As indicated above, we anticipated that the students will prefer native instructors at the very least for the Essay Writing course just for being a native speaker of English. Conversely, some students pointed to knowing and using Persian as an asset in teaching English. This was not the primary factor to convince most of the students to prefer non-native instructors, but the prevalence of its mention makes it worth noting. It seemed that occasional and limited use of Persian would give the students some relief from the anxiety inherent in learning English or a subject through English. Convincingly, Arash argued that, since non-native instructors are not perfect in English as the medium of instruction and communication, “they know that, sometimes, we cannot follow them and may get frustrated,” so they can also use Persian occasionally when it comes to sophisticated subject matter content or details of English language.

In view of gender, most of the students preferred male professors for both courses because they were perceived to be more competent and communicative. Those few students who selected female instructors thought that the female professors were fairer in grading and more understanding in interaction with students. They, including Mohamad, thought that female instructors usually treat everyone equally and respectfully in the class regardless of the sex, status, intelligence, and appearance of the students. They were much concerned about bias in assessment and discrimination in interaction and, rather passionately, thought that the female instructors are more sensitive to their impact on the academic engagement of their students.

Most students preferred male instructors since they evaluated more highly their competence and rapport with students. Comparing male and female instructors, they thought that male instructors are usually more competent and also confident about teaching. According to Zahra, for example, male instructors have a broad knowledge base, “they do not always go by the book and they often rely on their own experiences,” and can cope with problems in classes like “challenging questions.” Or based on Zahra’s personal experience, she notes that, “some of my [her] female instructors know a lot, but they do not seem as confident when teaching or when somebody comments or asks many questions.” In addition, male instructors were deemed to enjoy speech clarity as commented by Zahra and Nahid. In their views, they are likely to show more flexibility, understanding, and tolerance, even when students make mistakes; ask tricky questions; and share their stories, or, simply, in Ahmad’s words, “with them, I may get along very well.”

RQ3: How does perceived professors’ credibility affect their academic engagement, efforts, and success?

Instructor credibility was also investigated in terms of its impact on the academic engagement and achievement of the students. Overall, data analysis suggested that perceived instructor credibility would encourage students to put in more effort in their academic undertakings and to engage in class activities and course requirements. We looked into the responses and the emergent themes in relation to the instructor credibility components. For example, the students argued that, if their instructor is caring, they will feel more hopeful to succeed, work harder, and address their weaknesses and mistakes.

Caring instructors were thought to be more likely to observe the growth of their students to offer progressive advice. This was seen by Zohre to give them hope that, with the aid and care they receive, “they will be academically successful.” Similarly, for Arash, caring instructors are appreciated because they “motivate and encourage us to keep it up until we get what we want.” According to Ali and Zeynab, when someone is close, encouraging and more experienced with whom the students can share their problems and challenges, they will try harder not to disappoint their instructors. Students learn by trial and error; thus, when the students make errors or think to have certain weaknesses, they prefer to refer to instructors who are caring, Ahamd said.

It was also noted that some of the students had a tendency to listen or refer to instructors they thought are more competent. For example, Maryam commented that she asks her questions in the class or goes to the office of an instructor for assistance only if she is sure, through experience, that the instructor has something further to teach and she can help her get along. Competent instructors were viewed to be able to guide students and further point to extra resources, critical in higher education. Nahid and Zahra, associating the competence of the instructors with clarity of guidance and instruction, argued that such proficient instructors have various ways at hand to get the content across to the students, particularly when the medium of communication and instruction is a foreign language. They also contended that this understanding is the key to active engagement in classwork and ultimately academic success.

Since the respondents were EFL students, it did not surprise us that they frequently pointed to their objectives in learning English and how essential it is for an instructor to be competent or more specifically proficient in English. Through the analysis, this was most prevalent that they expected their instructors to set themselves as examples or models. An interesting comment was made by Shahla: “A proficient instructor indirectly tells me that if she could acquire English, why not? I can learn it as well.” Such instructors were also thought to be very helpful in providing ongoing exposure to English that is critical to learning English in an EFL context.

Finally, the students evaluated the influence of the character of the instructors, particularly instructor enthusiasm on their engagement and success. The interests and enthusiasm of the instructors are communicated through instructions and interaction. Ahmad stated that whether “instructors are interested in what they teach is clear and will interest me accordingly.” Shima and Mohamad also find energetic and excited instructors naturally attractive and motivating. It might be unrealistic to expect students to actively engage in classwork and academic assignments when the character of the instructors or class interaction is not encouraging enough, given the stressful nature of foreign langue learning contexts.

## Discussion

This study investigated the perceptions of the EFL university students of instructor credibility and its association with nativeness, gender, and subject matter. While instructor credibility is commonly characterized as a three-dimensional construct, involving competence, character, and caring (McCroskey and Teven, 1999), the data analysis generated a new component of performance in the context of EFL university education concerned with the effectiveness of classroom knowledge presentation and activity organization. It can be argued that the perception of credibility is relative to the views and interpretations of the students across different campuses and institutions (Hovland et al., 1953). Nonetheless, there are commonalities in the viewpoints acknowledged in the literature and supported in this study. For example, few conceptualizations or empirical studies did not take into consideration the competence of the instructors as a key component, although there has been variation in the interpretation of its importance and the implication it might have for the achievements of the students.

Among the credibility components, competence was most frequently cited by the participants and deemed as essential for credibility construction and learning enhancement. An EFL instructor was, therefore, assumed to be competent in pedagogical and linguistic knowledge areas in order to be able to provide clear, adequate, and current instructions to guide and facilitate the learning of the students. Because this study was conducted in a foreign language learning context, it was not surprising to see a variation in the perception of competence. Studies (e.g., Tucker, 1971; Wheeless, 1974a,b; Hendrix, 1997) have suggested that competence made the greatest contribution to the perceptions of the students of the instructor credibility; however, the perceptions as to what constructed competence varied as a function of different predispositions and preferences of the students for certain teaching behaviors. For example, in the study by Hendrix (1997), the participants described subject matter knowledge, teaching techniques, and expertise as the key areas of competence.

The students perceived the rapport of the instructors as an important part of the instructional/interpersonal process that also involves caring or having the best interests of the students in mind, empathy with the struggles and needs, support and encouragement, and closeness or immediacy of the students. Although in most experimental studies (e.g., Teven and McCroskey, 1997; McCroskey and Teven, 1999; Myers and Bryant, 2004; Teven and Hanson, 2004; Brann et al., 2005; Finn et al., 2009), caring was characterized as one dimension of tripartite classification of instructor credibility, in this study, it was eclipsed by and subsumed under the inclusive component of rapport. The instructor rapport, according to the students, can mitigate the effect of adverse psychological factors surrounding foreign language learning (e.g., anxiety). Foreign language anxiety, for example, can reduce when instructors create an understanding, friendly, and immediate environment in which students would be less afraid of making mistakes, which is one of the main causes of reticence and passivity of the L2 learners (for a discussion, see Cheng, 2000). The students frequently pointed to the anxiety in learning English, following instructions, and managing interaction both in a foreign language. L2 learning contexts are specifically associated with and affected by anxiety, which is argued to be different from the general trait anxiety (Gardner, 1985; MacIntyre and Gardner, 1989; Ellis, 1994; MacIntyre, 1998; Horwitz, 2001; Dörnyei, 2005). This apprehension involved in L2 communication, evaluation by others, and L2 assessment (Horwitz et al., 1986) can be aggravated by lack of teachers’ support (Tsui, 1996; Oxford, 1999; Young, 1999; Horwitz, 2001). This might be the reason rapport emerged as a significant credibility component.

The character associated with the personality traits and behaviors of the instructors, including courtesy, realisticness, humor, and enthusiasm, was also perceived to play a key role in instructor credibility and, in turn, in the achievements of the students. Although the two subcomponents of courtesy and realisticness are new to credibility theorizing, humor and enthusiasm have been acknowledged in other instructor credibility studies (see, for example, Wanzer et al., 2010; Wheeless et al., 2011; Keller et al., 2013; Myers et al., 2017; Bolkan et al., 2018). The students supposed that L2 learning classes need the respect of the instructors for the character of the students, which is usually at risk and a realistic demand noting that students learn variously at different paces. The lack of these two instructor attributes, however, would drive a wedge between students and instructors, and negatively impact the perceptions of the students of instructor credibility and their motivation to learn. Instructors’ humor was also perceived as important because it provides entertainment, reduces tension, and promotes motivation as also indicated in the literature (e.g., Cabello and Terrell, 1994; Dörnyei, 2001; Provine, 2002; Aboudan, 2009; Myers et al., 2017). The enthusiasm of the instructors was thought to reinforce the intent of the students to pursue educational studies. Benefits of the enthusiasm of the instructors have been linked to the motivation of the students and effective teaching in general education (Patrick et al., 2000; Witcher et al., 2001; Long and Hoy, 2006; Wheeless et al., 2011) and higher education (e.g., Jackson et al., 1999) as the focus of this study.

Instructor performance, incorporating activity-based instructions and classroom interaction, was also deemed as significant for producing greater perceptions of credibility. The frequency of comments on instructor performance led us to recognize that the classroom practice and performance of the L2 instructors, such as activity organization, task presentation, and preparation, affect the engagements of the students. Thus, noting the absence of “performance” in the credibility conceptualization, the results of this study induce that classroom performance of instructors also provokes the perceptions of the EFL students of the credibility of their instructors. From their points of view, possessing knowledge and skills is necessary but not enough. There should be actual use and reflection of what they know about their teaching to be more credible. As Hurt et al. (1978) put it, “there is, indeed, a difference between knowing and teaching, and that difference is communication in the classroom” (p. 3), which is essential for building credibility. This new component, emphasizing how an instructor performs in the classroom, is supported by the more recent literature on teacher quality such that measures have favored more output (i.e., performance) qualities than input qualities like certifications of teaching courses intended to equip teachers with knowledge bases (for a discussion, see Campbell et al., 2000; Caughlan and Jiang, 2014).

The study also examined the association of instructor credibility with a number of variables of interest (i.e., instructors’ gender, nativeness, and subject matter). The results revealed that, although most students consistently viewed native English-speaking instructors as generally more competent in subject matter and language, unexpectedly, only two students were willing to take “Research Methodology” and “Essay Writing” courses with these professors. The participants contented that native instructors enjoyed supremacy in the English language and subject matter knowledge because of their wide and first-hand linguistic experience and expertise. Their vast and flexible knowledge base in English also enables them to discuss the details of the content knowledge and to set real examples. These instructors, as also supported by the literature, are viewed to be providing a standard language model (Jieyin and Gajaseni, 2018; Okuda, 2019). The students who preferred these native instructors wished to improve the quality of their writing in the international proficiency tests, such as IELTS and TOFEL and, as demonstrated in the literature (Jieyin and Gajaseni, 2018), native English-speaking instructors were viewed to have the capability to provide authentic exposure that is essential for linguistic accuracy and fluency (Doerr, 2009; Tajeddin and Adeh, 2016) for the students preparing for international exams. Native instructors are also thought to be able to create a simulating learning environment that involves new hands-on practice and extracurricular activities, bringing variety and creativity to classroom learning (Gurkan and Yuksel, 2012). The advantages for native English-speaking instructors, however, were not viewed as highly in convincing the majority of the respondents to take the prospective courses with them.

Non-native Persian-speaking instructors were viewed by most of the respondents as more credible because of their rapport building and familiarity with the needs and challenges of the students. These instructors, according to the participants, understand the cultural and educational backgrounds of the students and their learning experiences. Having had similar experiences, non-native instructors are likely and willing to show an understanding of the shortcomings of the students in both Research Methodology and Essay Writing and their frustrations in learning a foreign language as also supported by Ma (2012). In addition, the participants referred to the ability of the non-native instructors to code-switch when they think the course content of instruction is hard to follow or they cannot make themselves understood. The L2-only position is associated with earlier language teaching methodologies like “The Direct Method” (Cook, 2001), mainly because of the advantages of exposing the L2 learners to only L2 input (MacDonald, 1993; Mattioli, 2004). More recently, however, cognitive and interactional advantages have been acknowledged for the feasible use of L1 (Brooks and Donato, 1994; Swain and Lapkin, 2000; Cook, 2001; Turnbull, 2001; Liu et al., 2004; Latsanyphone and Bouangeune, 2009). When the L2 was felt to be a barrier to knowledge and skills development, particularly while focusing on the nuances in university courses, the students in this study also preferred the prospective instructors to be able to resort L1 for the sake of content and details.

It is worth noting that, in a majority of teacher credibility studies, domestic and international professors were compared. Consistent with these studies (e.g., Rojas Gomez and Pearson, 1990), the findings from this study indicated that domestic professors (in this study, non-native instructors) were generally viewed as credible communicators of feelings and attitudes, more caring, empathetic and supportive of the students as compared with native English-speaking instructors. Studies (e.g., Bruce, 1986; Punyanunt-Carter et al., 2014; Zhang, 2014) highlighted the common challenges that international instructors and domestic students might both face in the classroom due to cultural assumptions and language barriers. They might upset effective teaching and learning, demotivate students, lower instructor-learner interaction, and ultimately decrease the students’ credibility perceptions of international instructors.

The results also indicated that, while only a few participants selected female instructors because of their fairness in interaction and grading practices, most of them preferred and viewed male professes as more competent and communicative due to their confidence, effective knowledge presentation, flexibility, and speech clarity. The study supports the findings by Hargett (1999), reporting the higher male instructor credibility, and conflicts with the study of Rojas Gomez and Pearson (1990), in which female instructors were found to be more competent, communicative, and credible than male counterparts. A common theme in the discussion of female and male instructors is the continuing tension in what distinguishes them and how they might be perceived by their students, given the historical, social, and cultural milieus (Clemmensen, 2002). How college students respond to or perceive their instructors differs and depends on how they are perceived by the respective instructors and how they are treated (Hoffmann and Oreopoulos, 2009). In this study, the preference of the students might be affected by their experience in a field, which is commonly dominated by male instructors in Iran.

An important issue in this study concerned how instructor credibility impacted the motivation, engagement, and success of the students. Perceived instructor credibility, according to the participants of the study, would drive them to put in more efforts in their academic study, engage in activities and classroom instruction, and face the learning challenges. The findings, hence, supported the argument that different dimensions of credibility engender different learning outcomes; for example, the instructor caring increases the effort and intention of the students to keep learning and furthering their education (Won et al., 2017), as well as their willingness to articulate their challenges and weaknesses (Muller, 2001). Instructor competence can improve the classroom engagement, knowledge retention, and learning of the students by enabling them to seek advice, raise questions, and complete assignments and in-class tasks (Frymier and Houser, 2000). Instructor enthusiasm was also found to affect the students’ motivation and interest in learning English. Instructor credibility, overall, produces positive outcomes that influence both the current decisions and intentions, and the future plans of the students.

In line with the findings of Myers et al. (2017), this study demonstrated that the participants valued a host of relational and rhetorical communicative behaviors (e.g., clarity and humor). Course-related behaviors of the instructors enhance the perceptions of the students of instructor credibility and promote entertainment and their learning, recall, and retention capabilities. The study, in other words, suggests that, in addition to demonstrating subject knowledge mastery and linguistic proficiency (rhetorical ethos), L2 instructors, if to attain credibility, should constantly ensure the interest and engagement of the students with the course content of instruction, thus relying on the relational ethos (for a discussion, see Mottet et al., 2006; Beebe and Mottet, 2009) or interpersonal dimension of the teacher–student interaction. Understanding the teachers of the needs and preferences of the students, and communication of caring and character are also components of this interpersonal and instructional interaction that assists the construction of credibility and learning.

Teaching, in effect, is described as a dual process involving advancing the content and relation. Effective teaching is thus equated with fostering interpersonal relationships between instructors and students through communicating character and caring, and fostering classroom learning through communicating knowledge competently (Frymier and Houser, 2000; Marzano and Marzano, 2003). It can be thus argued that instructor credibility and effectiveness are the results of instructor communication behaviors that lead to the learning outcomes of the students, including cognitive, affective, and behavioral achievements (McCroskey et al., 2004; Finn et al., 2009; Schrodt et al., 2009). These outcomes are most likely to be achieved through providing an environment in which the questions of the students are answered, their thoughts are shared, their voices are heard, and their feedback is welcomed (Schrodt et al., 2009). This, in turn, leads to a positive caring, interpersonal relationship and interaction between students and instructors (Brann et al., 2005).

## Conclusion

The study concludes that instructor credibility, in the context of EFL higher education, was viewed by the students to be a quadripartite construct, involving competence, rapport, caring, and performance. Discussing the findings, the study, in particular, highlighted the conceptualization of two components of “rapport” and “performance” and argued that, in the context of L2 learning, interpersonal relationship and in-class practice of the instructors have considerable significance, as they can reduce the anxiety and passivity associated with or resulting from L2 learning, and provoke classroom engagement of the students. The study also looked into the association of instructor credibility with gender, subject matter, and nativeness as instructional variables of interest. Interestingly, albeit being native meant being more competent in subject matters and proficient in L2, non-nativeness elicited more positive perceptions of credibility because of further perceived rapport building and caring, the qualities which male instructors were viewed to possess more than female counterparts. Another important conclusion, which can be drawn from this study, is that positive instructional outcome, such as motivation, engagement, persistence, and, ultimately, success imp on the perceptions of the students of the credibility of their instructors. Given the import of communication quality and instructor credibility, an overarching implication of the study findings for the context of EFL higher education is to encourage EFL instructors to enhance their ethos as an “instructional communication super-variable” (Teven and Katt, 2016, p. 184) by developing and sustaining rhetorical and relational skills.

## Limitations and Future Research

This qualitative study intended to investigate the voices and views of the university students to see what knowledge, behaviors, and skills characterize, enhance, or undermine instructor credibility in the classroom rather than those of researcher-imposed conceptualizations or operationalization. As such, we designed a scenario to conceptualize the findings in an EFL context and in relation to three variables of interest in higher EFL education. Furthermore, we sampled students from only one university in Iran. The perceptions of these students of the credibility of the EFL instructors might be affected by the cultural and instructional norms, and, as it is acknowledged in the literature, they may change during the education of the students (Hendrix, 1997; Schrodt et al., 2009). The nature of this qualitative study and the deliberate attempts made to contextualize it limits the generalization of the results, although we maintain that the understanding generated can be extrapolated to similar contexts. Future research can continue this line of investigation to employ other research methods or involve other important variables in other primary or higher education EFL contexts to heighten awareness and understanding about this critical concept.

## Data Availability Statement

The raw data supporting the conclusions of this article will be made available by the authors, without undue reservation.

## Ethics Statement

Ethical review and approval was not required for the study on human participants in accordance with the local legislation and institutional requirements. The patients/participants provided their written informed consent to participate in this study.

## Author Contributions

All authors listed have made a substantial, direct and intellectual contribution to the work, and approved it for publication.

### Conflict of Interest

The authors declare that the research was conducted in the absence of any commercial or financial relationships that could be construed as a potential conflict of interest.

## Appendix A: Scenario

**Please read the following before I ask you a few questions about your preferences:**

Suppose that the University is going to invite some visiting professors to teach some of your courses. You can choose from among native English-speaking (**from the same English-speaking country**) and non-native Persian-speaking teachers who are **female or male PhD holders** (assistant professors of applied linguistics). Their age range is from 40 to 45.

1. A female professor (First language: Persian)
2. A female professor (First language: English)
3. A male professor (First language: Persian)
4. A male professor (First language: English)

**Interview questions – teacher credibility:**

In general, what does instructor/professor credibility mean to you? What instructor/professor do you think is credible? Why?

For your course of writing, which professor do you think is most credible? Female/male, native/non-native professors. Why?

Does it make any difference if the course is “research”?

With which professor and in which course do you think you will get the highest score? Why?
